# Reliability and Validity of the Nigerian (Hausa) Version of the Stroke Impact Scale (SIS) 3.0 Index

**DOI:** 10.1155/2014/302097

**Published:** 2014-09-07

**Authors:** Ashiru Hamza Mohammad, Nabilla Al-Sadat, Siew Yim Loh, Karuthan Chinna

**Affiliations:** ^1^Center for Population Health, Department of Social and Preventive Medicine, Faculty of Medicine, University of Malaya, 50603 Kuala Lumpur, Malaysia; ^2^Department of Rehabilitation Medicine, Faculty of Medicine Building, University of Malaya, 50603 Kuala Lumpur, Malaysia; ^3^Julius Center, Department of Social and Preventive Medicine, Faculty of Medicine, University of Malaya, 50603 Kuala Lumpur, Malaysia

## Abstract

This study aims to test the translated Hausa version of the stroke impact scale SIS (3.0) and further evaluate its psychometric properties. The SIS 3.0 was translated from English into Hausa and was tested for its reliability and validity on a stratified random sample adult stroke survivors attending rehabilitation services at stroke referral hospitals in Kano, Nigeria. Psychometric analysis of the Hausa-SIS 3.0 involved face, content, criterion, and construct validity tests as well as internal and test-retest reliability. In reliability analyses, the Cronbach's alpha values for the items in Strength, Hand function, Mobility, ADL/IADL, Memory and thinking, Communication, Emotion, and Social participation domains were 0.80, 0.92, 0.90, 0.78, 0.84, 0.89, 0.58, and 0.74, respectively. There are 8 domains in stroke impact scale 3.0 in confirmatory factory analysis; some of the items in the Hausa-SIS questionnaire have to be dropped due to lack of discriminate validity. In the final analysis, a parsimonious model was obtained with two items per construct for the 8 constructs (Chi-square/df < 3, TLI and CFI > 0.9, and RMSEA < 0.08). Cross validation with 1000 bootstrap samples gave a satisfactory result (*P* = 0.011). In conclusion, the shorter 16-item Hausa-SIS seems to measure adequately the QOL outcomes in the 8 domains.

## 1. Introduction

The impact of stroke can be devastating, and unlike other disabling neurological conditions, stroke has a sudden onset leaving the individual and the family ill-prepared to deal with its residual impairments of physical, psychological, and social functions [1–3]. But until recently, stroke research in Nigeria has been largely focused on survival. The notion of quality of life (QOL) among researchers studying the consequence of stroke has gained increasing popularity in health care research following the increased need to improve the quality of lives saved. Recognition of variables related to life satisfaction is essential for such effort [4]. QOL is used as an outcome measure in clinical trials, in population studies, and in descriptive studies of patient groups.

QOL as a construct has been accepted as a multidimensional approach which covers the physical, functional, psychological, and social health dimensions and derived its theoretical framework based on modifications from the WHO's International Classification of Impairment, Disabilities, and Handicaps [5–9]. Instruments for the measurement of QOL can be generic or specific but are mainly developed in the English Language. They are often translated into other languages and validated for use in different cultures [10]. Many generic and disease specific health-status measurement instruments have been developed for the use in clinical trials evaluating various medical therapies, but their use in examining the impact of stroke and stroke interventions is limited [8, 11]. QOL data in stroke research will prove valuable in providing information and strategies to be utilized by health care providers and professionals in their attempt to improve the QOL of stroke patients. It may also be valuable in providing researchers/professionals with a global picture of recovery following stroke, development of more comprehensive rehabilitation interventions, resource allocation, policy formulation in a resource poor country, planning of rehabilitation services, and specific therapeutics.

While the use of outcome measures has generated increased interest in recent years [11, 12], the use of stroke specific measures such as the SIS 3.0 is also receiving a growing impetus in its use to evaluate QOL among stroke patients across cultures. Currently, there is no tool developed in Hausa language for the evaluation of QOL among stroke survivors. Since SIS 3.0 is written in the English Language, it is necessary to carry out linguistic validation of the MSPSS in Hausa language and further evaluate its psychometric properties so that it can be used in clinical research and practice in Nigeria.

## 2. Methods

The procedure involved in the development of the Hausa version of the SIS 3.0 involved linguistic translation which was published elsewhere [13] and the subsequent testing and psychometric validation of the Hausa-SIS 3.0. These cognitive processes are deemed important to make sure that the Hausa-SIS 3.0 was measuring the same concept as the original English version. This is important if comparisons are to be made on the QOL outcomes between different cultures.

This study was conducted at three stroke referral hospitals that are dedicated to the rehabilitation and neurologic disorders in Kano, Nigeria: Aminu Kano Teaching Hospital, Murtala Mohammad Specialist Hospital, and Mohammad Abdullahi Wase Specialist Hospital. Patients with a diagnosis of stroke who were admitted or seen as outpatients during the subacute stage of recovery at the neurology and physiotherapy clinics in these three hospitals between December 2010 and January 2012 were included in the study.

## 3. Description of the Survey Instrument

The stroke impact scale (SIS) version 3.0 (Supplementary Appendix 1, see Supplementary Material available online at http://dx.doi.org/10.1155/2014/302097) is a stroke specific measure of QOL that has undergone extensive psychometric testing and reported excellent internal consistency, with Cronbach's alpha values ranging from 0.93 to close to 1 [14–18]. SIS 3.0 was developed based on the viewpoint, perception, position, and contributions of stroke patients, caregivers, and health professionals with stroke expertise [19]. It is a psychometrically robust 59-item stroke-specific self-report measure developed to assess a number of dimensions of QOL. It consists of 8 domains: Strength, Hand function, Mobility, Physical and instrumental activities of daily living (ADL/IADL), Memory and thinking, Communication, Emotion, and Social participation. Scores for each domain range from 0 to 100, where higher scores indicate better QOL. The items in Strength subscale measure physical strength. The items in Memory and thinking, Communication, ADL/IADL, Mobility, and Hand function subscales measure the level of difficulty. Items in the Emotion and Social Participation subscales are rated in terms of frequency. The SIS 3.0 also includes a question (item 60) to assess the patient's global perception of recovery. The respondent is asked to rate his or her percentage of recovery on a visual analogue scale of 0 to 100, with 0 meaning no recovery and 100 meaning full recovery. Past studies indicate substantial improvement in most SIS domains in patients recovering from mild and moderate stroke [20, 21].

The translated Hausa version of the SIS 3.0 [13] was administered among 35 stroke patients and they were reassessed a week after the first evaluation. The data collected were used in reliability and test-retest reliability analyses. This preliminary test was done by face-to-face interviews with the patients in order to acquire remarks and suggestions on the Hausa scale.

Subsequently, the psychometric properties of the Hausa-SIS was tested using a sample of 140 stroke surviving patients, aged between 35 to 82 years old, selected randomly from the three stroke referral hospitals in Kano, Nigeria. Besides testing the questionnaire, the feasibility of administering the questionnaire under field conditions was also noted. In the confirmatory factor analysis (CFA), the AMOS version 18 software was used to test the instrument validity. AMOS 18 software enables specification, estimation, assessment, and presentation of models to show hypothesized relationship among variables. The software helps build models accurately than multivariate techniques. It provides structural equation modeling, uses Bayesian analysis to improve estimate of model parameter, and offers various models to create different data sets. CFA categorically tests a priori hypotheses about relations between observed variables and latent variables or factors [22, 23].

Approval for the conduct of this study was obtained from the Medical Ethics Committee of the University of Malaya (Eth. Comm./IRB Reference number 830.7), Ministry of Health Kano state, Nigeria (HMB/GEN/488/11), and Aminu Kano Teaching Hospital, Nigeria (AKTH/MAC/SUB/12A/P3/IV/801).

## 4. Data Analysis

The psychometric properties of the Hausa-SIS were tested through content validity, face validity, criterion validity, construct validity, and internal consistency. Double data entry was carried out and cross-checked to assure the consistency and quality of the data. Most analyses were carried out using the statistical program SPSS for Windows version 20.0. Descriptive statistics were obtained for demographic variables, means, and standard deviations for continuous variables and frequency and percentages for categorical variables. The face and content validity were tested through a pilot test involving stroke patients during the linguistic validation process by the experts in quality of life measures, Public Health, and stroke rehabilitation. The criterion validity of the Hausa-SIS is defined as the performance of the instrument as compared to the existing gold standard or outcome that the measure was intended to assess [24]. Confirmatory factor analysis (CFA) was performed using AMOS version 18 to determine if the number of factors and the loadings measured (indicator) variables on them conform to what is expected on the basis of preestablished theory [24]. In CFA the 8 constructs with their respective indicators were tested simultaneously.

## 5. Results

All the 140 stroke survivors who were selected agreed to participate. There were 73 females and 67 males in the study and their ages ranged from 35 to 82 years with a mean age of 57.7 ± 13.8 years. In the sample, cerebral ischemic stroke was detected in 48 (34.3%) patients, and cerebral haemorrhage was in 11 (7.9%) and could not be determined (indeterminate) in 81 (57.9%) patients. 63 (45.0%) patients had left hemispheric lesions, whereas 77 (55.0%) had affected right hemisphere. In the sample 107 (76.4%) were urban residents.

Content and face validity testing of the Hausa-SIS was performed by the experts to ensure that the Hausa-SIS had achieved conceptual, semantic, and operational equivalence with the original index. During translation and where required, expressions used in the items were subjected to more culturally acceptable linguistic equivalents similar to concept and meaning to the original items. A pretesting and pilot study with 30 stroke survivors additionally attested the face and content validation of the Hausa-SIS. The time taken to answer the questionnaire was also acceptable, that is, 15–20 minutes. The feasibility of administering the instrument under field condition was also verified. The instrument was found to be easily conceivable, simple, clear, and appropriate for the assessment of QOL among this group of stroke survivors.

In reliability analyses, the Cronbach's alpha values for the items in Strength, Hand function, Mobility, ADL/IADL, Memory and thinking, Communication, Emotion, and Social participation domains were 0.80, 0.92, 0.90, 0.78, 0.84, 0.89, 0.58, and 0.74, respectively.

**Figure 1:**
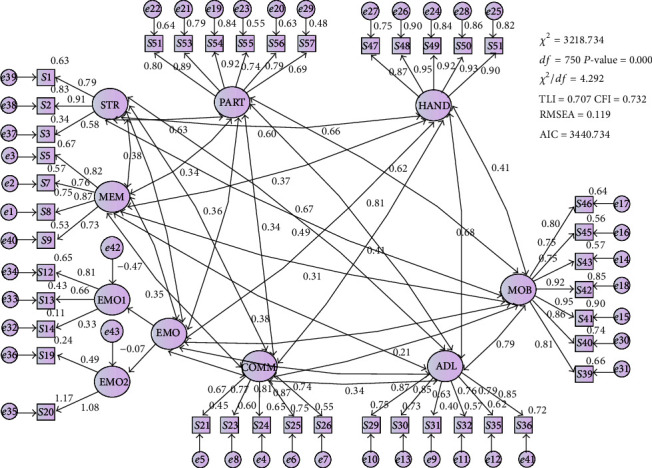
Initial tested model.

In CFA, the 8-factor model (Figure 1) did not fit well (Chi-square/df > 3, TLI and CFI < 0.9, and RMSEA > 0.08). Guided by modification indices option in AMOS, the model was corrected shortcoming in discriminant validity.

**Figure 2:**
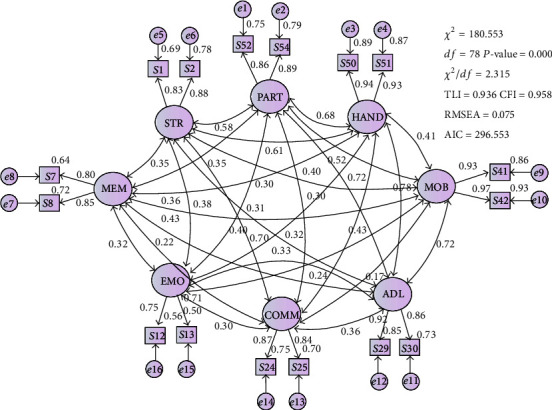
The final model.

In the final analysis, a parsimonious model (Figure 2) was obtained with two items per construct for the 8 constructs (Chi-square/df < 3, TLI and CFI > 0.9 and RMSEA < 0.08).

The unstandardized regression weights and the factor loadings (standardized regression weights) are shown in Table 1.

All the factor loadings are more than 0.7 and the average variance extracted (AVE) for all the constructs are more than 0.50. The pairwise correlation coefficients between the constructs are provided in Table 2.

Since the highest correlation value is less than 0.85, there is no sign of multicollinearity [25]. Further test on discriminant validity was done by comparing the AVE and the *R*-squared values between constructs, pairwise. There is sufficient discriminant validity ids the AVEs are more than the *R*-square value [26]. Based on the results that are summarized in Table 3, there is sufficient discriminant validity between the constructs.

In Table 3, the average variance extracted (AVE) values are more than 0.5. Thus, there is sufficient convergent validity within the construct. When compared pairwise, all the AVEs are higher than the *R*-squared values. Thus, there is sufficient discriminant validity between the constructs.

## 6. Discussion

The principal results from the investigation of the SIS 3.0 with stroke survivors in Kano, Nigeria suggest that the SIS 3.0 is a suitable measure for assessing quality of life among stroke survivors. The SIS 3.0 validation was conducted in close collaboration between the translation committee comprising of the multiprofessional experts from University of Malaya, Bayero University Kano, Nigeria and professional translators from the Freedom Radio Nigeria* muryar jama'a 99.5FM* (an independent radio), and the copyright owner/developers of the questionnaire. During translation and where required, expressions used in the items were subjected to more culturally acceptable linguistic equivalents similar to concept and meaning to the original items.

During pilot study, the respondents did not encounter problems with understanding the contents of the Hausa version of the SIS 3.0 but raised concern on some of the terms used in the items. They provided some suggestions and a consensus was reached and we retained the items by providing supplementary explanatory sentences. The instrument was found to be easily conceivable, simple, clear, and appropriate for the assessment of QOL among this group of stroke survivors. In the reliability analysis, the cronbach's alpha values for the items in the respective 8 domains were above 0.55. This indicates that the Hausa-SIS 3.0 index items are well correlated with one another in a decisive way and are suitable to constitute an index. In the CFA, the initial tested model did not fit well (Chi-square/df > 3, TLI and CFI < 0.9 and RMSEA > 0.08). After correcting for lack of discriminant validity, the 8 domains were maintained but reduced to two items each. The model fit in the final model was good (Chi-square/df < 3, TLI and CFI > 0.9, and RMSEA < 0.08).

To our knowledge, this study is the first of its kind conducted in Nigeria that involved translating and testing the psychometric properties of the Hausa version of the SIS 3.0. In accordance with the proposal by Clark and Watson, the number of items in an instrument determines the sample size to be used in the psychometric testing. For an instrument with 20 items or less, a sample size between 100 and 200 subjects is deemed to be adequate [25, 26]. Hence, in this study the sample size of 140 is considered to be adequate for testing the psychometric properties of the Hausa-SIS 3.0 index.

The strength of our study was the fact that the study involved rigorous forward-backward translations of the tool. An extensive AMOS modelling to confirm the factor structure, and in the end we obtained a good validated instrument. The study had some limitations; firstly, although we have a good sample size, it would have been better with a bigger sample size. Secondly, the measure was not tested against any measure of quality of life including disability and dependence in the activities of daily living. Analysis of the metric properties for severity groups is also absent. This needed to be conducted in further studies.

## 7. Conclusion

It was found that the 16-item Hausa-SIS 3.0 (Supplementary Appendix 2) which is the Hausa short version of the SIS 3.0 seems to measure adequately the QOL outcomes in the 8 domains. Such researchers investigating quality of life in stroke survivors could use this instrument for studies with the hope that it will prove valuable in providing information and strategies to be utilized by health care providers and professionals in their attempt to improve the QOL of stroke patients.

## Figures and Tables

**Table 1:** Regression weights.

			Unstandardized estimate	S.E.	C.R.	*P*	Standardized estimate
S50	←	HAND	1.000				.943
S51	←	HAND	1.190	.053	22.466	∗∗∗	.934
S1	←	STR	1.000				.830
S2	←	STR	1.038	.081	12.761	∗∗∗	.882
S8	←	MEM	1.000				.850
S7	←	MEM	.821	.058	14.144	∗∗∗	.798
S41	←	MOB	1.000				.927
S42	←	MOB	.961	.043	22.166	∗∗∗	.967
S30	←	ADL	1.000				.857
S29	←	ADL	1.143	.062	18.402	∗∗∗	.922
S25	←	COMM	1.000				.837
S24	←	COMM	1.043	.125	8.331	∗∗∗	.869
S13	←	EMO	1.000				.709
S12	←	EMO	1.111	.115	9.637	∗∗∗	.746
S52	←	PART	1.000				.863
S54	←	PART	.963	.066	14.651	∗∗∗	.891

**Table 2:** Covariance and correlation.

			Covariance	S.E.	C.R.	*P*	Correlation
PART	↔	HAND	.833	.111	7.504	∗∗∗	.676
HAND	↔	MOB	.714	.131	5.443	∗∗∗	.415
HAND	↔	COMM	.474	.093	5.103	∗∗∗	.433
HAND	↔	EMO	.280	.075	3.749	∗∗∗	.324
ADL	↔	COMM	.463	.108	4.283	∗∗∗	.359
COMM	↔	EMO	.242	.074	3.273	.001	.301
STR	↔	MEM	.299	.072	4.145	∗∗∗	.350
PART	↔	MEM	.384	.092	4.176	∗∗∗	.346
HAND	↔	MEM	.316	.083	3.808	∗∗∗	.299
STR	↔	EMO	.265	.065	4.066	∗∗∗	.380
PART	↔	EMO	.282	.081	3.491	∗∗∗	.313
PART	↔	COMM	.341	.094	3.615	∗∗∗	.298
STR	↔	ADL	.779	.109	7.136	∗∗∗	.696
ADL	↔	EMO	.334	.091	3.694	∗∗∗	.329
PART	↔	ADL	1.040	.139	7.475	∗∗∗	.718
HAND	↔	ADL	1.015	.127	7.990	∗∗∗	.732
MOB	↔	ADL	1.470	.187	7.840	∗∗∗	.725
MEM	↔	MOB	.558	.123	4.517	∗∗∗	.360
MOB	↔	EMO	.307	.106	2.898	.004	.243

MOB	↔	COMM	.273	.120	2.271	.023	.171
MEM	↔	ADL	.536	.105	5.092	∗∗∗	.430
MEM	↔	COMM	.217	.081	2.684	.007	.221
MEM	↔	EMO	.247	.072	3.441	∗∗∗	.317
PART	↔	STR	.578	.092	6.288	∗∗∗	.582
HAND	↔	STR	.577	.085	6.794	∗∗∗	.608
STR	↔	MOB	.561	.112	4.986	∗∗∗	.404
STR	↔	COMM	.356	.078	4.556	∗∗∗	.403
PART	↔	MOB	.933	.151	6.189	∗∗∗	.519

**Table 3:** Test of discriminant validity.

	HAND	STR	MEM	MOB	ADL	COMM	EMO	PART
HAND	0.881							
STR	0.370	0.733						
MEM	0.089	0.123	0.680					
MOB	0.172	0.163	0.130	0.897				
ADL	0.536	0.484	0.185	0.526	0.792			
COMM	0.187	0.162	0.049	0.029	0.129	0.728		
EMO	0.105	0.144	0.100	0.059	0.108	0.091	0.530	
PART	0.457	0.339	0.120	0.269	0.516	0.089	0.098	0.769

Values in parenthesis are AVEs and the others are the pair-wise *R*-squared values.
